# Effect of laser applications with different agents on dentin surface roughness, bacterial adhesion and tubule occlusion

**DOI:** 10.1007/s00784-025-06443-w

**Published:** 2025-06-25

**Authors:** Ayşe Dündar, Kaan Yılmaz, Çağatay Barutçugil, Özlem Koyuncu Özyurt

**Affiliations:** 1 Department of Restorative Dentistry, Faculty of Dentistry, Akdeniz University, Antalya, 07058 Turkey; 2 Edirne Ağız ve Diş Sağlığı Merkezi, Edirne, Turkey; 3 Department of Medical Microbiology, Faculty of Medicine, Akdeniz University, Antalya, Turkey

**Keywords:** Dentin sensitivity, Surface roughness, Bacterial adhesion, Laser, Sodium fluoride, CPP-ACP

## Abstract

**Objective:**

This study aimed to evaluate the effects of laser application with different agents on dentin surface roughness, tubule occlusion, and bacterial adhesion in vitro.

**Materials and methods:**

A total of 216 dentin blocks from bovine incisors were divided into nine groups. Treatments included 5% sodium fluoride (NaF) varnish, casein phosphopeptide–amorphous calcium phosphate (CPP-ACP), Er, Cr: YSGG laser, and diode laser in various combinations. Surface roughness (SR) was measured using a profilometer. *S. mutans* and *S. mitis* suspensions were incubated for 24 h, and bacterial adhesion was quantified (×10⁸ Cfu/ml) and visualized using confocal laser scanning microscopy. Tubule occlusion was assessed via SEM imaging and analyzed with ImageJ software. Statistical analysis was performed using one-way ANOVA and Tukey HSD (*p* = 0.05).

**Results:**

Except for the CPP-ACP groups treated with laser, all groups exhibited significantly higher surface roughness (*p* < 0.05); however, this did not influence bacterial adhesion (*p* > 0.05). All treatments significantly reduced the number and diameter of open dentinal tubules (*p* < 0.05). The combination of NaF with Er, Cr: YSGG and diode laser enhanced tubule occlusion, while CPP-ACP with laser had no additional effect.

**Conclusions:**

Increased surface roughness, except in CPP-ACP with laser groups, did not affect bacterial adhesion. All desensitization methods effectively promoted tubule occlusion, with the diode laser demonstrating greater efficacy than the Er, Cr: YSGG laser.

**Clinical significance:**

Lasers should be used with caution as they may increase the roughness of the dentin surface. The combined application of NaF with lasers may improve its clinical efficacy.

## Introduction

Dentin hypersensitivity (DH) is a common clinical condition characterized by sharp, short-term pain arising from exposed dentin in response to thermal, tactile, osmotic, or chemical stimuli [1]. The prevalence of DH ranges between 4% and 57% in the general population and being higher in individuals with periodontal disease [2].

According to Brännström’s hydrodynamic theory, external stimuli cause the movement of fluid inside the dentinal tubules, activating mechanoreceptors and resulting in the sensation of pain [3]. The number and diameter of open dentinal tubules have been demonstrated to directly correlate with DH [4]. Exposure of dentinal tubules can occur due to several factors such as enamel loss, gingival recession, abrasion, attrition, erosion and periodontal disease [5]. Acidic diets, gastric reflux and mechanical wear also contribute to the exposing and widening of dentinal tubules, thereby increasing dentin permeability and sensitivity [6]. Methods such as permeability test, mineral alteration assessment, and SEM image analysis are used to evaluate the efficacy of desensitization methods in vitro [1, 6, 7]. However, measuring the number and diameter of open dentinal tubules using SEM images enables both visual and quantitative assessments to be made.

The management of DH for pain relief primarily focuses on occluding open dentinal tubules to reduce fluid movement or desensitizing nerve responses. Various products are available for in-office desensitisation including gel, solution, varnish, resin sealers, glass ionomers and dentin adhesives [8]. Fluoride varnishes, which contain high concentrations of fluoride, can form a mechanical barrier on exposed dentin. Clinically, 5% sodium fluoride (NaF) is used to treat dentin hypersensitivity. Topical applications of NaF facilitate the deposition of calcium fluoride (CaF₂) on the tooth surface, effectively occluding open dentinal tubules and thereby reducing dentin permeability [6, 9]. Casein phosphopeptide (CPP) is a milk-derived phosphoprotein capable of stabilizing high concentrations of calcium and phosphate ions in solution under acidic and basic pH conditions. This is due to CPP binding to calcium and phosphate to form stable complexes known as casein phosphopeptide-amorphous calcium phosphate (CPP-ACP).

 [10]. CPP-ACP has been shown to be effective in remineralizing enamel. However, the existing literature on its use in remineralizing dentin or treating dentin hypersensitivity is very limited. Several clinical studies have demonstrated its effectiveness in alleviating sensitivity issues; however, it has also been noted that the duration of this effect is brief [11, 12]. At this point, it is thought that the combined application of NaF and CPP-ACP with laser will increase its clinical effectiveness [13]. Many theories have been proposed to explain the effectiveness of laser application in the treatment of DH. These theories include sealing dentinal tubules by melting and recrystallizing dentine, evaporating dentinal fluid, suppressing nerve conduction to provide an analgesic effect, and obliterating dentinal tubules with tertiary dentine formation [14, 15]. When the dentin surface is irradiated with a laser, some of the laser energy is transmitted to the dentin [16], potentially altering its morphology. Further investigation is needed to establish whether combining lasers with other agents will reduce surface roughness. Additionally, the literature on the use of lasers in combination with desensitizers is limited. This is the first study to quantitatively evaluate the roughness, bacterial adhesion, tubule diameter, and number of open tubules of the dentin surface when desensitizing agents are applied with laser.

This study aims to evaluate the effects of 5% sodium fluoride varnish, CPP-ACP, the Er, Cr: YSGG laser, and the diode laser, both alone and in combination, on dentin surface roughness, bacterial adhesion, and tubule occlusion. The null hypotheses tested in this context are as follows:

1. The change in surface roughness of dentin after the desensitization methods are applied is similar to that of the control group.
2. The number of *S. mutans* and *S. mitis* adhering to the dentin surface after the desensitization methods are applied is similar to that in the control group.
3. The number and the diameter of open dentinal tubules following the desensitization methods are similar to those in the control group.

## Materials and methods

### Preparation of dentin samples

Freshly extracted bovine incisors were stored in 0.1% thymol solution at a pH of 7.0 for one month. The crowns were separated from the roots with a cutting device (IsoMet 1000, Buehler Ltd., Lake Bluff, IL) using a diamond disc. The roots were individually mounted on the cutting machine, and 216 buccal dentin blocks (5 × 5 × 3 mm) were obtained from them. To standardize the root dentin substrate, the root dentin specimens were flattened and sanded using water-cooled sandpaper (600-, 800-, 1200-, and 2000-grit) under standard conditions. A 9.0 mm² area was marked on the outer surface of each specimen on which the treatments were performed.

### Measurement of surface roughness

A gel containing 19% EDTA (ethylenediamine tetraacetic acid, MD-ChelCream, Meta-Biomed Co., Ltd., Korea) was applied to the exposed surface of all samples. After one minute, each sample was washed in distilled water. This procedure was designed to simulate the pattern of exposed dentin by opening the dentinal tubules.

A profilometer (Surftest SJ-201, Mitutoyo, Tokyo, Japan) was used to analyse the surface of the dentin samples in two dimensions, and the surface roughness (Ra) of each sample was recorded in micrometres (µm). The needle diameter of instrument tip was 5 μm. Measurements were taken at 2.5 mm intervals on the sample and at a speed of 0.5 mm/s. Roughness was measured from three different areas of each sample: two lateral areas and one central area. The arithmetic mean of the obtained values was calculated. The profilometer was calibrated before and after taking measurements in each group.

### Experimental groups

The following materials and methods were employed in the present study: 5% sodium fluoride varnish (NaF, Enamelast Fluoride Varnish, Ultradent, USA); CPP-ACP (MI Paste Plus, GC Corporation, Tokyo, Japan); 2780 nm wavelength Er, Cr: YSGG laser (Waterlase, Biolase, California, USA); and a 940 nm wavelength diode laser (Biolase, California, USA). Following the measurement of the initial surface roughness, the 216 dentin samples were randomly divided into nine groups (*n* = 24) and subjected to the following procedures:

Group 1 (G1): It was not subjected to any form of treatment and thus served as the control group.

Group 2 (G2): The specimens were dried using cotton and 9.0 mg of 5% NaF varnish was applied for 10 s before being left for 2 min. The specimens were then immersed in distilled water at 37 °C for 24 h. The residual varnish was then removed after 24 h using a gauze swab.

Group 3 (G3): CPP-ACP was applied using an applicator in accordance with the manufacturer’s instructions. The CPP-ACP was left for 5 min before being removed from the tooth surface with a cotton pellet. The tooth surface was then rinsed with distilled water.

Group 4 (G4): The specimens were treated with an Er, Cr: YSGG laseroperating at a power of 0.25 W and a repetition rate of 20 Hz. The laser was operated at a pulse duration of 140 µs for 30 s using a Z6 sapphire tip (600 μm in diameter, 6 mm in length) at a distance of 1 mm without the presence of air or water.

Group 5 (G5): The specimens were irradiated (333.4 J/cm^2^ energy density) using a high-intensity diode laser in non-contact continuous mode, with the laser positioned at a distance of 1 mm from the tissue and a power output of 1.5 W. The root dentin surface was scanned for 20 s (10 s horizontally and 10 s vertically) using 400-µm-diameter optical fiber. The irradiation was performed manually to simulate clinical practice.

Group 6 (G6): The specimens were treated with 9.0 mg of 5% NaF varnish for 10 s. After a waiting period of 2 min, the specimens were immersed in distilled water at 37 °C for 24 h. The residual varnish was then removed using a gauze pad and the Er, Cr: YSGG laser was applied in accordance with the protocol previously outlined.

Group 7 (G7): The CPP-ACP was applied to the tooth surface using an applicator, left for 5 min, then removed with a cotton pellet and rinsed with distilled water. The Er, Cr: YSGG laser was then applied as described.

Group (G8): 9 mg of 5% NaF varnish were applied for 10 s. Following a 2-minute wait, the specimens were immersed in distilled water at 37 °C for 24 h. After 24 h, the varnishes was removed with a cotton pellet and the diode laser was applied as described.

Group (G9): The CPP-ACP was applied to the tooth surface using an applicator. After 5 min, it was removed with a cotton pellet and rinsed with distilled water. Then the diode laser was applied as described.

The final surface roughness of the dentin samples was measured using a profilometer (Surftest SJ-201, Mitutoyo, Tokyo, Japan) following the application of desensitizing methods, as previously described.

### Bacterial adhesion

This study utilized *Streptococcus mutans* and *Streptococcus mitis* bacteria as model organisms. These bacteria were obtained from the National Type Culture Collection of the Turkish Ministry of Health, Refik Saydam Hygiene Center, and the Department of Microbiology at Akdeniz University Faculty of Medicine. A total of 108 specimens were used in the experiment, with 6 samples allocated to each of the two bacterial strains and 12 samples to each of the nine experimental groups. Prior to bacterial adhesion, the dentin samples were sterilized in an autoclave (Tomy Model SX-700E Autoclave, Tokyo, Japan) at 121 °C for 15 min.

Firstly, an artificial saliva solution was prepared to form a pellicle layer on the dentin samples and for bacterial adhesion purposes. The following formula was used to prepare two liters of artificial saliva [17]:

8.4 mg of sodium fluoride (NaF), 2,560 mg of sodium chloride (NaCl), 332.97 mg of calcium chloride (CaCl₂), 250 mg of magnesium chloride hexahydrate (MgCl₂·6 H₂O), 189.48 mg of potassium chloride, 0.1 ml of hydrogen triphosphate (H3PO4) (85%), and 0.1 mmol of sodium hydroxide. All ingredients were mixed in a magnetic stirring device until the mixture was clear. The pH of the saliva was then measured using a pH meter. The pH was adjusted to a range of 6.5–7.0 by adding 30 ml of 0.1 M NaOH to the mixture.

The artificial saliva was sterilized in an autoclave (Tomy Model SX-700E Autoclave, Tokyo, Japan). Then, 140 mg of Type II mucin (Sigma-Aldrich Chemie GmbH, Deisenhofen, Germany) was added to each 100 ml of artificial saliva.

One packet of ready-to-use phosphate buffered saline (PBS) (Sigma, USA) with a pH of 7.4 was added to 1 L of distilled water, and the mixture was thoroughly agitated to prepare a 0.01 M PBS solution. The solution was then sterilized in an autoclave (Tomy Model SX-700E Autoclave, Tokyo, Japan) at 121 °C for 15 min.

The composition of Brain-Heart Infusion (BHI**)** Broth (Neogen Culture Media, UK) is as follows:

BHI 17.5 (12.5–5 g), tryptose (10 g), glucose (2 g), sodium chloride (5 g), and disodium hydrogenate (2.5 g). The medium was prepared by adding 37 g of BHI to one liter of distilled water, adjusting the pH to 7.4, and then adding 37 g of 5% glucose. The mixture was then sterilized in an autoclave (Tomy Model SX-700E Autoclave, Tokyo, Japan) at 121 °C for 15 min.

The composition of the blood agar medium used to grow bacteria is as follows: beef extract (10 g/L), balanced peptone (10 g/L), sodium chloride (5 g/L), agar (12 g/L), pH 7.4 ± 0.2, and defibrinated sheep blood (5%).

The lyophilized *S. mutans* strain was subjected to a series of steps to facilitate culturing. Firstly, the strain was opened and inoculated into a liquid medium of 5% sucrose BHI (Neogen culture media, UK). It was then placed in an incubator (Thermo/Forma Thermo Fisher Scientific CO_2_ Water Jacketed Incubator, South Carolina USA) at 35 ˚C with 5% CO_2_ for 3–5 days to allow growth. Next, it was inoculated onto prepared blood agar (Neogen culture media, UK). The *S. mitis* strains were thawed from a -80 °C stock and inoculated onto sheep blood agar. These were then incubated for 24 h in an aerobic, 5% CO_2_ incubator (Thermo/Forma Thermo Fisher Scientific CO_2_ Water Jacketed Incubator, South Carolina, USA) at 35 °C. The colonies were then transferred to tubes containing 5 ml of 5% sucrose BHI liquid medium. These tubes were then placed in an incubator set at 35 °C with 5% CO_2_ for a 24-hour incubation period.

After 24 h, the tubes were placed in a centrifuge (Zentrifuge Rotofix 32 Hettich, Tuttlingen, Germany) for 5 min. Subsequently, 5 ml of PBS (Multicell, Canada) was added to each tube, and the tubes were subjected to the centrifugal process one more for 15 s. A bacterial suspension with a concentration of 10^8^ colony-forming units (Cfu) was then prepared from the sediment that had accumulated at the bottom of the tube.

The samples, which had been sterilized by autoclaving, were transferred to sterile petri dishes. Then, 5 ml of saliva and mucin mixture was added to each dish. The dishes were placed in an incubator set at 37 °C for 1 h with the aim of forming a pellicle. Once this process was completed, the dishes were washed with 5 ml of saline and transferred to new sterile petri dishes. Then, 200 µL of the prepared bacterial suspension was added to the surface of each sample and left for 15 min. Then, 5% sucrose BHI liquid medium was added to each petri dish to cover the sample. The petri dishes were then incubated in a 35˚C incubator (Thermo/FormaThermo Fisher Scientific CO_2_ Water Jacketed Incubator, USA) with 5% CO_2_ for 24 h to allow bacterial adhesion.

Samples were collected 24 h after completion of bacterial adhesion into tubes containing 2 ml of PBS each and vortexed for 60 s with a vortex device (Scilogex MX-S Vortex Mixer, Scilogex, LLC, USA). 200 µL of BHI liquid medium was added to each well of the 96-well microplate. Then, 20 µL of the washing solution from the vortexed samples was added. The microplates were placed in an incubator set at 35 °C with 5% CO_2_ for 24 h. The microplates were then analyzed using an automated microplate reader (Robonik-Readwell Touch Automatic ELISA Plate Analyzer, Maharashtra, India). The optical densities were measured at a wavelength of 630 nm. All procedures were carried out separately for each bacterium.

### Confocal laser scanning microscopy (CLSM) analysis

After bacterial adhesion was complete, the samples were fixed to the slides with nail varnish, ensuring the desensitized surfaces faced upwards.

Confocal laser scanning microscopy was used to evaluate the The LIVE/DEAD BacLight Viability Kit ((L13152) Invitrogen Molecular Probes, Eugene, OR). This kits consist of the green, fluorescent nucleic acid dye SYTO 9 and the red fluorescent nucleic acid dye propidium iodide. When used on its own, the SYTO 9 stain usually penetrates all the bacteria in the population, including those with intact and damaged membranes. In contrast, propidium iodide stains only bacteria with damaged membranes. Therefore, when used in combination, bacteria with intact cell membranes are stained fluorescent green, and bacteria with damaged membranes were stained fluorescent red. The LIVE/DEAD BacLight Kit (L13152) includes two transfer pipettes (Pipette A and Pipette B) and a pre-prepared dye mixture of SYTO 9 and propidium iodide dyes.

LIVE/DEAD BacLight dye solution was prepared by mixing the contents of both pipette A (containing yellow-orange dye) and pipette B (containing purple dye) with 5 mL of distilled water. The dye mixture was then used to stain the entire surface of each specimen mounted on the slides (average of 2 µl). The slides were kept in the dark for 15 min at room temperature. The specimens were then rinsed with 5 ml of sterile saline to remove any dye residue.

The samples were visualized using a confocal laser scanning microscope (Zeiss Lsm 510 Meta, Zeiss GmbH, Jena, Germany).

### SEM analysis for tubule occlusion

The surface morphology of dentin was observed using scanning electron microscopy (SEM). Following the treatment protocols, the dentin samples were immersed in artificial saliva (pH 7.2) at room temperature for 24 h, rinsed with distilled water, and air-dried. The samples were coated with a 5-nm gold layer in a sputter coater and then placed in the vacuum chamber of the SEM at an accelerating voltage of 10 kV. SEM images of each dentin sample were taken to examine the morphological changes at ×2000 magnification. The number of open tubules was determined using the Image J program, in a manner consistent with previous study [18]. Calculations were performed using Image J software version 1.47 s (NIH, Bethesda, MD, USA) with grey pixel differences between the inside and outside of dentin tubules defined by color filters. All analyses were performed by the same experienced examiner. The total number and diameter of open tubules in each image were calculated for all samples.

### Statistical analysis

Statistical analyses were conducted using the SPSS 22 software program (IBM Corp., Armonk, NY, USA). One-way analysis of variance (ANOVA) and Tukey HSD tests were employed to assess the differences between the groups. An independent sample t-test was utilized to make between-group comparisons. The significance level for all results was set at *p* = 0.05.

## Results

### Surface roughness

The mean values and standard deviations of the changes in surface roughness (Ra) of the samples are shown in Table 1. As a result of the statistical analyses made in this study, the roughness change of the G1 group, where the desensitization method was not applied, and the G7 and G9 groups were found to be similar (*p* < 0.05). However, the roughness change in the G1 group was significantly lower than that in the other groups (*p* < 0.05), which showed similar results in terms of roughness change (*p* < 0.05).

**Table 1:** Mean and standard deviation of surface roughness change (Ra), *S. Mutans* and *S. Mitis* adhesion, number of open dentin tubules and open dentin tubule diameter (µm)

Groups	Desensitization Method	Roughness Change (µm)	S. Mutans (10^8^ Cfu/ml)	S. Mitis (10^8^ Cfu/ml)	Number of Open Tubules	Tubule Diameter (µm)
G1	Control	0.00 ± 0.012^a^	2.35 ± 1.93^a^	8.45 ± 3.88^a^	101.33 ± 21.00^a^	1.90 ± 0.18^a^
G2	NaF	0.19 ± 0.25^b^	2.51 ± 1.14^a^	7.16 ± 6.80^a^	26.66 ± 7.90^bc^	1.49 ± 0.25^b^
G3	CPP-ACP	0.17 ± 0.16^b^	2.58 ± 0.66^a^	4.64 ± 3.95^a^	32.16 ± 6.20^bc^	1.57 ± 0.13^bc^
G4	Er, Cr: YSGG	0.16 ± 0.11^b^	3.28 ± 1.34^a^	5.35 ± 4.66^a^	46.16 ± 4.30^b^	1.67 ± 0.12^bc^
G5	Diode	0.24 ± 0.09^b^	5.45 ± 2.90^a^	6.01 ± 1.83^a^	28.00 ± 6.60^bc^	1.22 ± 0.17^d^
G6	NaF + Er, Cr: YSGG	0.22 ± 0.19^b^	2.46 ± 1.34^a^	2.98 ± 1.45^a^	31.83 ± 4.40^bc^	1.32 ± 0.16^cd^
G7	CPP-ACP + Er, Cr: YSGG	0.09 ± 0.06^ab^	4.01 ± 2.20^a^	4.56 ± 2.64^a^	43.33 ± 5.00^b^	1.41 ± 0.12^cd^
G8	NaF + Diod	0.16 ± 0.10^b^	3.74 ± 2.48^a^	6.28 ± 1.71^a^	22.00 ± 3.80^c^	1.24 ± 0.21^d^
G9	CPP-ACP + Diod	0.09 ± 0.07^ab^	5.42 ± 2.38^a^	9.30 ± 8.39^a^	47.2 ± 5.80^b^	1.50 ± 0.15^bcd^

According to one-way analysis of variance and Tukey HSD results, different lowercase letters in each column indicate statistically different groups. (*p* < 0.05)

### Bacterial adhesion

The mean and standard deviation values for adhesion of *S. mutans* and *S. mitis* are presented in Table 1. No statistically significant difference was observed in the number of *S. mutans* and *S. mitis* bacteria in any of the groups (*p* > 0.05). All groups had similar bacterial adhesion.

**Fig. 1:**
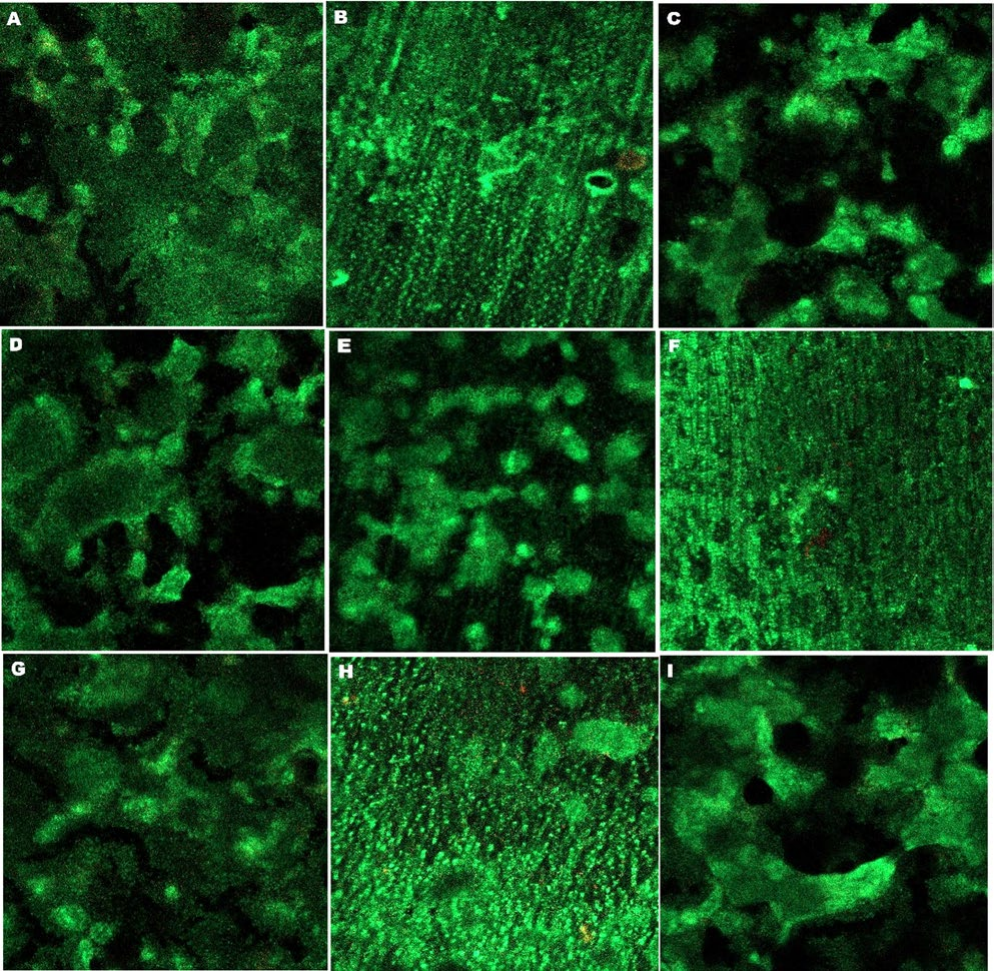
Representative CLSM images of dentin samples from each *S. mutans*-adhered group. The images correspond to the following groups: control (**A**), NaF (**B**), CPP-ACP (**C**), Er, Cr: YSGG (**D**), Diod (**E**), NaF + Er, Cr: YSGG (**F**), CPP-ACP + Er, Cr: YSGG (**G**), NaF + Diod (**H**), and CPP-ACP + Diod (**I**)

**Fig. 2:**
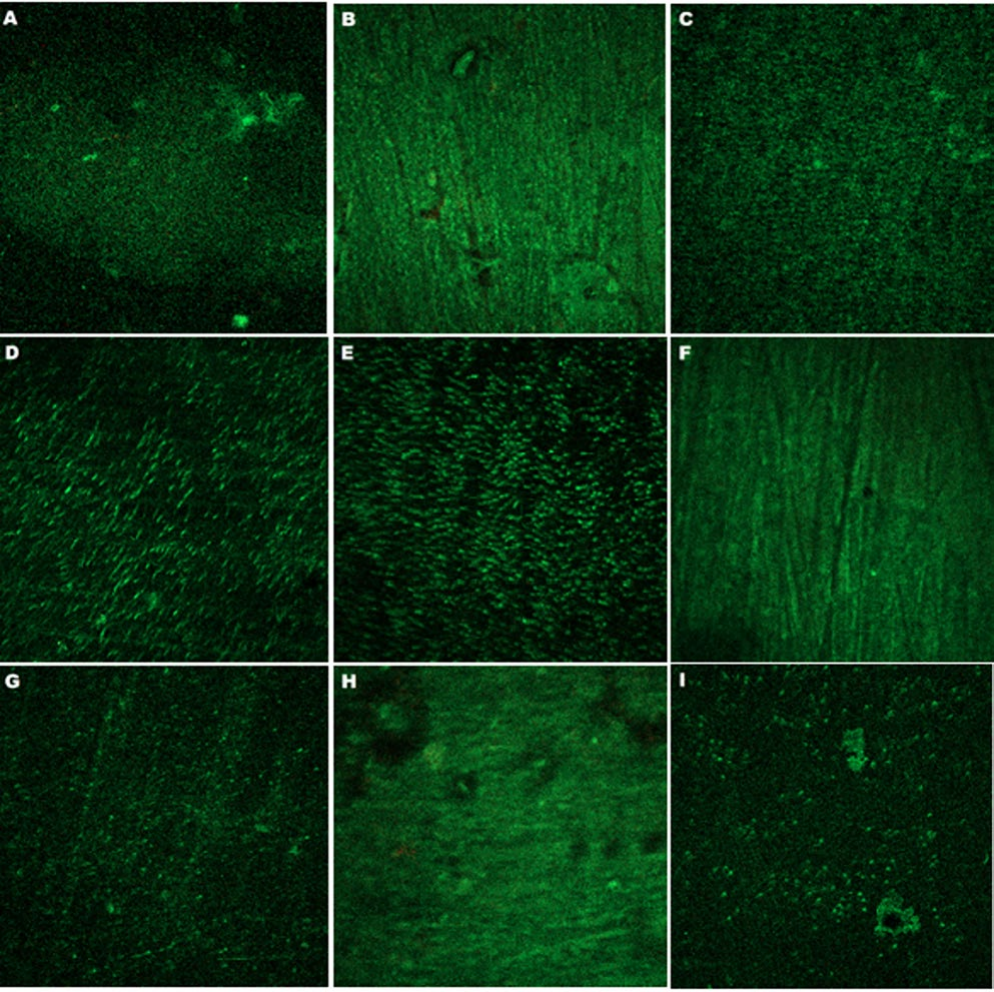
Representative CLSM images of dentin samples from each *S. mitis*-adhered group are. The images correspond to the following groups: control (**A**), NaF (**B**), CPP-ACP (**C**), Er, Cr: YSGG (**D**), Diod (**E**), NaF + Er, Cr: YSGG (**F**), CPP-ACP + Er, Cr: YSGG (**G**), NaF + Diod (**H**), and CPP-ACP + Diod (**I**)

CLSM examination revealed mature, multilayered biofilm structures on all dentin specimens (see Figs. 1 and 2). Live cells appear green and dead cells appear red. Generally, a higher prevalence of live cells was observed on the surfaces of the specimens. Notably, among the *S. mutans* samples examined, those from the NaF-treated groups exhibited a slight red staining pattern (Fig. 1B, F and H). Another notable observation in the NaF-treated groups was that *S. mutans* adhesion occurred in diffuse small clusters rather than in large aggregates (Fig. 1B, F and H).

### Tubule occlusion

The number of open dentinal tubules and tubule diameters were calculated from the SEM images of the specimens using the Image J program. The mean values and standard deviations for the number of open dentinal tubules and tubule diameters of the specimens are shown in Table 1. Statistical analysis revealed significant differences between the groups (p˂0.05).

Statistical evaluations revealed that the number of open dentinal tubules for all desensitizing methods was significantly lower than in the control group (*p* < 0.05). Additionally, the dentinal tubule diameter of all groups was found to be significantly narrower than that of the control group (*p* < 0.05). The combination of NaF with Er, Cr: YSGG, and diode lasers significantly enhanced in the effectiveness of NaF in reducing tubule diameter (*p* < 0.05). Conversely, the combined application of CPP-ACP and a laser did not significantly impact tubule occlusion (*p* > 0.05). Furthermore, the diode laser was found to be more effective than the Er, Cr: YSGG laser at narrowing the tubule diameter.

**Fig. 3:**
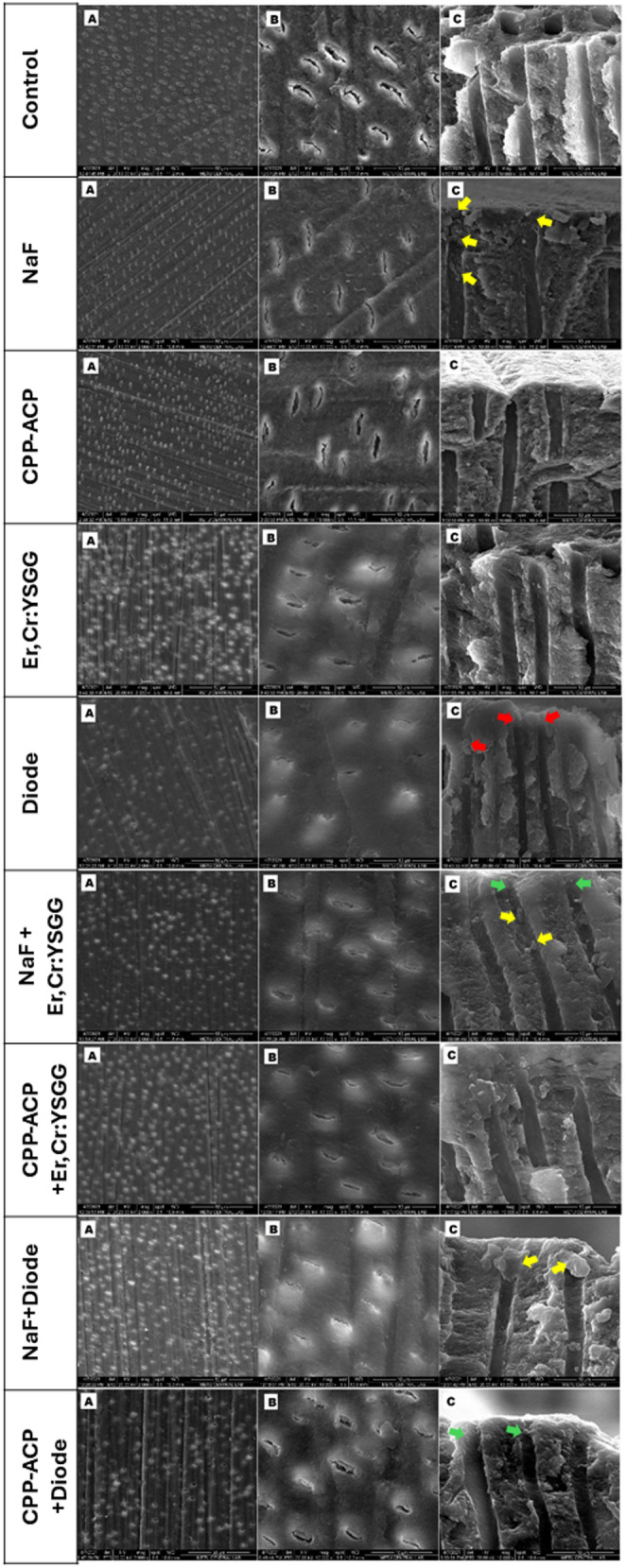
Representative SEM images of specimens. A and B correspond to SEM images of the buccal surfaces of the groups, and C shows the interface. (Yellow arrow: tubule plug, red arrow: narrowing and occlusion of the tubule orifice due to ablation, green arrow: narrowing of the tubule orifice)

Analysis of the SEM images revealed that most of the dentinal tubules in the control group were open, with a larger tubule diameter observed in images A and B compared to the study groups (Fig. 3). Most of the tubules in the study groups, particularly in G2, G5 and G8, were found to be completely closed. In the open tubules, a marked narrowing was observed. Image from the interface section reveal the presence of plugs at the tubule orifice in NaF-treated groups (Fig. 3, yellow arrow). Image of the diode laser group shows that the tubule orifice has narrowed and become occluded due to ablation (Fig. 3, red arrow). The CPP-ACP group exhibited narrowing and occlusion in most of the tubules. The diameter of the open dentinal tubules was slightly smaller than that of the control group. It is noteworthy that the tubule diameters were slightly narrower in the groups in which the Er, Cr: YSGG laser was used in combination with either NaF or CPP-ACP compared to the group in which the Er, Cr: YSGG laser was used alone (Fig. 3 images A and B).

Of particular interest are the observations of narrowing of the tubule orifice (Fig. 3 green arrow) and obstructions in the tubule (Fig. 3 yellow arrow) in the group where the Er, Cr: YSGG laser was used in combination with NaF. In the group where the CPP-ACP was applied with the diode laser, plugs were observed in the tubule orifice (see Fig. 3, image B), as well as narrowing of the tubule orifice (see Fig. 3, green arrow).

## Discussion

This study is significant in evaluating the effects of laser treatment combined with different desensitizing agents on dentin surface roughness, bacterial adhesion, and dentinal tubule occlusion. A salient finding of the study is that while all desensitizing methods reduced the diameter and number of dentinal tubules, the diode laser and the diode laser combined with NaF were more effective. Another noteworthy observation was that, although surface roughness was generally higher in all groups than in the control group, this had no effect on bacterial adhesion.

The variability in dentin permeability is considerably less in bovine teeth than in human teeth [19]. The significantly higher age range for tooth extraction in humans can potentially introduce variations in dentin texture. These variations may cause problems when trying to ensure standardization in the study. In addition to being widely regarded as the optimal substitute for human teeth in laboratory-based studies [19, 20], the decision to use bovine teeth in this study was also based on the importance of having a standardised dentin surface on which to measure the number and diameter of open dentinal tubules.

Laser applications are used to treat DH by melting, recrystallization, and occluding tubules on the dentin surface [14, 15]. Since these procedures alter the surface morphology of dentin, they may increase surface roughness and lead to increased bacterial adhesion and caries formation [21, 22]. The results of our study demonstrated that both the Er, Cr: YSGG laser and the diode laser caused a greater increase in surface roughness when applied alone, except in the groups where they were applied in combination with CPP-ACP. The first null hypothesis is rejected, as the surface roughness change in the non-laser groups is also higher than in the control group. A recently published study evaluated the surface roughness and bacterial adhesion of different lasers used for dentin sensitivity treatment [23]. The study found that only the Er, Cr: YSGG laser increased the surface roughness. This finding is consistent with our own study. On the contrary, the roughness remained constant in the group treated with the 980 nm diode laser. The discrepancy observed between the two studies may be due to methodological differences. Specifically, the diode laser output power used by the researchers’ study (0.5 W) was substantially lower than that used in our study (1.5 W). In another study that employed the same diode laser parameters as in our own, it was observed that the surface of the dentin treated with the diode laser exhibited greater roughness compared to the untreated surfaces, a finding that aligns with our own observations [24]. Despite the observed increase in surface roughness resulting from laser applications in our study, it is evident that roughness has no influence on the adhesion of *S. mutans* and *S. mitis*. In this context, the second null hypothesis that was tested in the study is accepted. The experimental groups did not demonstrate an increase in bacterial adhesion when compared to the control group for either bacterium. These findings are consistent with those of other studies documented in the existing literature [23–25]. Bacterial adhesion is expected to increase after the surface roughness exceeds a threshold value of 0.2 μm [26]. In our study, similar bacterial adhesion in all groups can be explained by the fact that the highest roughness value (0.24 μm) was close to this threshold value. However, in addition to evaluating the effect of only the Ra parameter on bacterial adhesion, it would be useful to evaluate more parameters, such as free surface energy and contact angle, with further studies. As a matter of fact, there are findings in the literature that surface roughness alone has no effect on bacterial adhesion, but high surface free energy and low contact angle values increase bacterial adhesion [27, 28]. In the present study, although the roughness change of the control group was very low, bacterial adhesion was found to be similar to the study groups. This leads to the idea that the above-mentioned parameters other than roughness may have an effect on bacterial adhesion.

The efficacy of desensitizing applications was assessed by measuring the number and diameter of open dentinal tubules across all experimental groups. The results showed that these parameters had decreased compared to the control group. This suggests that all the methods examined in the study were effective in occluding tubules. Consequently, the third null hypothesis tested in the study should be rejected.

One of the groups tested in this study was 5% NaF varnish, which has a proven effect in the treatment of dentin sensitivity [29]. This effect occurs when NaF interacts with calcium ions in the dentin fluid to form calcium fluoride (CaF₂) crystals, which accumulate on the dentin tubules and occlude the tubule orifice. However, the dimensions of these crystals (approximately 0.05 μm) may reduce the effectiveness of a single NaF application in reducing the diameter of dentinal tubules, potentially requiring repeated treatments [30]. To overcome this limitation, it has been proposed that NaF be used in combination with laser systems [13]. The findings of this study are consistent with these results. Applying NaF alone or in combination with Er, Cr: YSGG and the diode lasers did not significantly affect the number of dentinal tubules. However, the co-application of NaF and lasers did cause a significant narrowing of the tubule diameter (Fig. 3). Recent studies have also demonstrated that applying of a laser in combination with NaF leads to an increase in tubule obstruction [6, 31].

Due to the high collagen content of dentin, remineralization occurs via a different mechanism than that of enamel. CPP stabilizes amorphous calcium phosphate nanoclusters that can localize in demineralized zones and deliver calcium and phosphate ions into the collagen matrix [32]. The CPP-ACP is thought to prevent demineralization and enhance remineralization by increasing the concentration of calcium and phosphate ions, leading to a state of supersaturation [33]. Treatment of the dentin surface with CPP-ACP paste results in the formation of a layer that fills the intratubular spaces [32] or epitaxial growth of hydroxyapatite crystals remaining in the collagen fibrils [34]. The CPP-ACP has been demonstrated to reduce DH by promoting mineral deposition and obstructing dentinal tubules. A number of clinical and laboratory studies have been conducted to test its efficacy in treating of DH, but the results are controversial [11, 12, 35, 36]. The present study found that CPP-ACP application was more effective than the control group at obstructing tubules. The results of the study were consistent with those observed in the NaF group. The study was designed to simulate the short-term effect of desensitization. It is important to note that active cycles in the oral cavity, such as long-term application or brushing simulation with acid attacks, were not included in the study. In studies where CPP-ACP has been found to be effective in treating of DH or occluding tubules, it has been applied repeatedly and over extended periods [11, 36]. To increase the efficacy of CPP-ACP, it has been tested in combination with Er, Cr: YSGG and diode laser. However, the findings of the current study demonstrate that the co-application of CPP-ACP with laser does not demonstrate superiority in tubule occlusion compared to single application. The most significant outcome of the combined CPP-ACP and laser treatment was that the surface roughness was lower than in the other groups and comparable to that of the control group.

From a mechanistic standpoint, Er, Cr: YSGG lasers operate at 2780 nm, with high affinity for water and hydroxyapatite, resulting in superficial ablation and melting of dentin. Diode lasers (940 nm), on the other hand, penetrate deeper and may stimulate reparative dentinogenesis [37]. These lasers are classified as high-intensity lasers. The heat generated by the high-intensity laser causes the dentin to melt and then re-solidify after cooling [38]. This melting leads to coagulation, protein precipitation, or the formation of insoluble calcium complexes in the dentin tissue, which occlude the dentinal tubules [23]. Additionally, the heat produced by the lasers may stimulate the pulpal tissue to form tertiary dentin [37]. This biological response contributes to tubule occlusion from within the pulp-dentin complex and reduces sensitivity. In the present study, the number of open dentinal tubules was comparable in both laser groups, but notably lower than in the control group. However, the tubule diameter was narrower in the diode laser group than in the Er, Cr: YSGG laser group. In a randomized controlled split-mouth trial in which both lasers were used to treat dentin hypersensitivity, the Er, Cr: YSGG was found to be more effective [39]. Although the parameters of the Er, Cr: YSGG laser used in the aforementioned study were the same as those used in our own investigation, the output power and operating time of the diode laser were considerably lower. This discrepancy can be attributed to the reduced energy transfer associated with lower output power. This discrepancy may explain the differences between the two studies.

In groups where lasers were used in combination with agents, similar values were observed for tubule diameters in all groups, while noteworthy differences were seen in the number of open dentinal tubules. Using NaF with a diode laser resulted in the occlusion of more tubules than using CPP-ACP with lasers. This finding is consistent with the results of previous studies [40, 41]. Clinically, the use of NaF varnish in combination with a diode laser may be more successful in treating dentine sensitivity.

The objective of this study was to examine the effect of desensitization treatments on dentin surface roughness, and dentinal tubules in a laboratory setting. It is not possible to examine these in vivo. The absence of acid attacks and tooth brushing simulation are limitations of this study.

## Conclusions

This study investigated the combination of two lasers and two different desensitizing agents. The results obtained from this study are as follows:

All groups exhibited an increase in dentin surface roughness, except those groups that underwent CPP-ACP application combined with lasers. However, this increased surface roughness did not result in increased *S. mutans* and *S. mitis* adhesion. In all desensitizing treatments, the number of open dentinal tubules and tubule diameter were lower than in the control group. Furthermore, the combination of NaF with Er, Cr: YSGG and diode laser increased the effectiveness of NaF in reducing tubule diameter. However, the co-application of CPP-ACP with the laser had no significant impact on tubule occlusion. The 940 nm diode laser was found to be more effective than the 2780 nm Er, Cr: YSGG laser at narrowing the tubule diameter.

## Data Availability

No datasets were generated or analysed during the current study.

## References

[1] Dundar A, Yavuz T, Orucoglu H, Daneshmehr L, Yalcin M, Sengun A (2015) Evaluation of the permeability of five desensitizing agents using computerized fluid filtration. Niger J Clin Pract 18(5):601–606. 10.4103/1119-3077.158949 26096236 10.4103/1119-3077.158949

[2] Favaro Zeola L, Soares PV, Cunha-Cruz J (2019) Prevalence of dentin hypersensitivity: systematic review and meta-analysis. J Dent 81:1–6. 10.1016/j.jdent.2018.12.015 30639724 10.1016/j.jdent.2018.12.015

[3] Brännström M, Aström A (1972) The hydrodynamics of the dentine; its possible relationship to dentinal pain. Int Dent J 22(2):219–227 4505631

[4] Khoubrouypak Z, Hasani Tabatabaei M, Chiniforush N, Moradi Z (2020) Evaluation of the effects of 810 Nm diode laser alone and in combination with Gluma(©) and chromophore on dentinal tubule occlusion: A scanning Electron microscopic analysis. J Lasers Med Sci 11(3):268–273. 10.34172/jlms.2020.45 32802286 10.34172/jlms.2020.45 PMC7369560

[5] Demirci M, Karabay F, Berkman M et al (2022) The prevalence, clinical features, and related factors of dentin hypersensitivity in the Turkish population. Clin Oral Investig 26(3):2719–2732. 10.1007/s00784-021-04245-4 35083586 10.1007/s00784-021-04245-4

[6] Okur E, Eyüboğlu GB (2022) Evaluation of dentin tubule plugging efficiencies and effects on dentin surface roughness of dentin desensitizing agents, the er,cr:ysgg laser, and their combination after Erosion-abrasion cycles: an in vitro study. Oper Dent 47(1):E35–e51. 10.2341/21-086-l 35289911 10.2341/21-086-L

[7] Dilber E, Malkoc MA, Ozturk AN, Ozturk F (2013) Effect of various laser irradiations on the mineral content of dentin. Eur J Dent 7(1):74–80 23407579 PMC3571513

[8] Liu XX, Tenenbaum HC, Wilder RS, Quock R, Hewlett ER, Ren YF (2020) Pathogenesis, diagnosis and management of dentin hypersensitivity: an evidence-based overview for dental practitioners. BMC Oral Health 20(1):220. 10.1186/s12903-020-01199-z 32762733 10.1186/s12903-020-01199-z PMC7409672

[9] Ritter AV, de Miguez LDW, Caplan P, Swift DJ Jr (2006) Treating cervical dentin hypersensitivity with fluoride varnish: a randomized clinical study. J Am Dent Assoc 137(7):1013–1020 quiz 1029. 10.14219/jada.archive.2006.0324 16803829 10.14219/jada.archive.2006.0324

[10] Cross KJ, Huq NL, Palamara JE, Perich JW, Reynolds EC (2005) Physicochemical characterization of casein phosphopeptide-amorphous calcium phosphate nanocomplexes. J Biol Chem 280(15):15362–15369. 10.1074/jbc.M413504200 15657053 10.1074/jbc.M413504200

[11] Ayan G, Mіsіllі T, Buldur M (2025) Home-use agents in the treatment of dentin hypersensitivity: clinical effectiveness evaluation with different measurement methods. Clin Oral Investig 29(1):63. 10.1007/s00784-025-06155-1 39810073 10.1007/s00784-025-06155-1 PMC11732919

[12] Kowalczyk A, Botuliński B, Jaworska M, Kierklo A, Pawińska M, Dabrowska E (2006) Evaluation of the product based on recaldent technology in the treatment of dentin hypersensitivity. Adv Med Sci 51(Suppl 1):40–42 17458057

[13] Ipci SD, Cakar G, Kuru B, Yilmaz S (2009) Clinical evaluation of lasers and sodium fluoride gel in the treatment of dentine hypersensitivity. Photomed Laser Surg 27(1):85–91. 10.1089/pho.2008.2263 19182972 10.1089/pho.2008.2263

[14] Yilmaz HG, Bayindir H (2014) Clinical and scanning electron microscopy evaluation of the er,cr:ysgg laser therapy for treating dentine hypersensitivity: short-term, randomised, controlled study. J Oral Rehabil 41(5):392–398. 10.1111/joor.12156 24602082 10.1111/joor.12156

[15] Umana M, Heysselaer D, Tielemans M, Compere P, Zeinoun T, Nammour S (2013) Dentinal tubules sealing by means of diode lasers (810 and 980 nm): a preliminary in vitro study. Photomed Laser Surg 31(7):307–314. 10.1089/pho.2012.3443 23756100 10.1089/pho.2012.3443

[16] Meng Y, Huang F, Wang S et al (2023) Evaluation of dentinal tubule occlusion and pulp tissue response after using 980-nm diode laser for dentin hypersensitivity treatment. Clin Oral Investig 27(8):4843–4854. 10.1007/s00784-023-05114-y 37382717 10.1007/s00784-023-05114-y

[17] Aykent F, Yondem I, Ozyesil AG, Gunal SK, Avunduk MC, Ozkan S (2010) Effect of different finishing techniques for restorative materials on surface roughness and bacterial adhesion. J Prosthet Dent 103(4):221–227. 10.1016/s0022-3913(10)60034-0 20362765 10.1016/S0022-3913(10)60034-0

[18] Kim JS, Han SY, Kwon HK, Kim BI (2013) Synergistic effect of dentinal tubule occlusion by nano-carbonate apatite and CO2 laser in vitro. Photomed Laser Surg 31(8):392–397. 10.1089/pho.2012.3470 23822167 10.1089/pho.2012.3470

[19] Schmalz G, Hiller KA, Nunez LJ, Stoll J, Weis K (2001) Permeability characteristics of bovine and human dentin under different pretreatment conditions. J Endod 27(1):23–30. 10.1097/00004770-200101000-00007 11487159 10.1097/00004770-200101000-00007

[20] Teruel Jde D, Alcolea A, Hernández A, Ruiz AJ (2015) Comparison of chemical composition of enamel and dentine in human, bovine, Porcine and ovine teeth. Arch Oral Biol 60(5):768–775. 10.1016/j.archoralbio.2015.01.014 25766469 10.1016/j.archoralbio.2015.01.014

[21] Candan M, Ünal M (2021) The effect of various asthma medications on surface roughness of pediatric dental restorative materials: an atomic force microscopy and scanning electron microscopy study. Microsc Res Tech 84(2):271–283. 10.1002/jemt.23584 32905650 10.1002/jemt.23584

[22] Guler S, Unal M (2018) The evaluation of color and surface roughness changes in resin based restorative materials with different contents after waiting in various liquids: an SEM and AFM study. Microsc Res Tech 81(12):1422–1433. 10.1002/jemt.23104 30295386 10.1002/jemt.23104

[23] Parlar Oz O, Karagozoglu İ, Kocer I, Demırkol N, Zer Y (2024) The effect of laser therapy for the treatment of dentin hypersensitivity on surface roughness and bacterial adhesion. Lasers Med Sci 39(1):212. 10.1007/s10103-024-04166-0 39120679 10.1007/s10103-024-04166-0 PMC11315743

[24] Cury MS, Silva CB, Nogueira RD, Campos MGD, Palma-Dibb RG, Geraldo-Martins VR (2018) Surface roughness and bacterial adhesion on root dentin treated with diode laser and conventional desensitizing agents. Lasers Med Sci 33(2):257–262. 10.1007/s10103-017-2356-x 29032514 10.1007/s10103-017-2356-x

[25] Yuanhong L, Zhongcheng L, Mengqi L, Daonan S, Shu Z, Shu M (2016) [Effects of nd: YAG laser irradiation on the root surfaces and adhesion of Streptococcus mutans]. Hua Xi Kou Qiang Yi Xue Za Zhi 34(6):579–583. 10.7518/hxkq.2016.06.006 28318157 10.7518/hxkq.2016.06.006 PMC7030872

[26] Bollen CM, Lambrechts P, Quirynen M (1997) Comparison of surface roughness of oral hard materials to the threshold surface roughness for bacterial plaque retention: a review of the literature. Dent Mater 13(4):258–269 11696906 10.1016/s0109-5641(97)80038-3

[27] Bilgili D, Dündar A, Barutçugil Ç, Tayfun D, Özyurt ÖK (2020) Surface properties and bacterial adhesion of bulk-fill composite resins. J Dent 95:103317. 10.1016/j.jdent.2020.103317 32165185 10.1016/j.jdent.2020.103317

[28] Bilgili Can D, Dündar A, Barutçugil Ç, Koyuncu Özyurt Ö (2021) Evaluation of surface characteristic and bacterial adhesion of low-shrinkage resin composites. Microsc Res Tech 84(8):1783–1793. 10.1002/jemt.23735 33586287 10.1002/jemt.23735

[29] Minkov B, Marmari I, Gedalia I, Garfunkel A (1975) The effectiveness of sodium fluoride treatment with and without iontophoresis on the reduction of hypersensitive dentin. J Periodontol 46(4):246–249. 10.1902/jop.1975.46.4.246 1055218 10.1902/jop.1975.46.4.246

[30] Gaffar A (1998) Treating hypersensitivity with fluoride varnishes. Compend Contin Educ Dent 19(11):1088–1090 1092, 1094 passim 10202463

[31] Vazirizadeh Y, Lawaf S (2022) Comparison of the efficacy of 940-nm diode laser, gluma, and 5% sodium fluoride varnish in dentinal tubule occlusion. Lasers Dent Sci 6:1–8. 10.1007/s41547-021-00139-6

[32] Poggio C, Lombardini M, Vigorelli P, Ceci M (2013) Analysis of dentin/enamel remineralization by a CPP-ACP paste: AFM and SEM study. Scanning 35(6):366–374. 10.1002/sca.21077 23427062 10.1002/sca.21077

[33] Nongonierma AB, Fitzgerald RJ (2012) Biofunctional properties of caseinophosphopeptides in the oral cavity. Caries Res 46(3):234–267. 10.1159/000338381 22572605 10.1159/000338381

[34] Zhou Z, Ge X, Bian M et al (2020) Remineralization of dentin slices using casein phosphopeptide-amorphous calcium phosphate combined with sodium tripolyphosphate. Biomed Eng Online 19(1):18. 10.1186/s12938-020-0756-9 32245476 10.1186/s12938-020-0756-9 PMC7119276

[35] Yu J, Yi L, Guo R, Guo J, Yang H, Huang C (2021) The stability of dentin surface biobarrier consisting of mesoporous delivery system on dentinal tubule occlusion and Streptococcus Mutans biofilm Inhibition. Int J Nanomed 16:3041–3057. 10.2147/ijn.s290254 10.2147/IJN.S290254 PMC8088303 33948084

[36] Kijsamanmith K, Banomyong D, Burrow MF et al (2019) Effect of conventional and acid-modified casein Phosphopeptide-Amorphous calcium phosphate Crèmes on dentin permeability before and after acid challenge. Oper Dent 44(5):530–535. 10.2341/17-382-l 30951440 10.2341/17-382-L

[37] Guler C, Alan H, Demir P, Vardi N (2020) Effects of diode laser irradiation on dental pulps in rats. Bratisl Lek Listy 121(4):293–296. 10.4149/bll_2020_046 32356445 10.4149/BLL_2020_046

[38] Rosa RR, Calazans FK, Nogueira RD, Lancellotti AC, Gonçalves LS, Geraldo-Martins VR (2016) Effects of different desensitizing treatments on root dentin permeability.Braz Oral Res 30(1):e111. 10.1590/1807-3107BOR-2016.vol30.0111 10.1590/1807-3107BOR-2016.vol30.0111 27737364

[39] Pourshahidi S, Ebrahimi H, Mansourian A, Mousavi Y (2019) Comparison of er,cr:ysgg and diode laser effects on dentin hypersensitivity: a split-mouth randomized clinical trial. Clin Oral Investig 23(11):4051–4058. 10.1007/s00784-019-02841-z 30778688 10.1007/s00784-019-02841-z

[40] Cakar G, Kuru B, Ipci SD, Aksoy ZM, Okar I, Yilmaz S (2008) Effect of er:yag and CO2 lasers with and without sodium fluoride gel on dentinal tubules: a scanning electron microscope examination. Photomed Laser Surg 26(6):565–571. 10.1089/pho.2007.2211 19099386 10.1089/pho.2007.2211

[41] Tunar OL, Kuru BE (2022) Microstructural changes of human dentin tubules after citric acid immersion of specimens treated with different desensitising approaches: an SEM analysis. Oral Health Prev Dent 20:369–378. 10.3290/j.ohpd.b3464895 36259440 10.3290/j.ohpd.b3464895 PMC11640751

